# Physician Use of Electronic Health Records: Survey Study Assessing Factors Associated With Provider Reported Satisfaction and Perceived Patient Impact

**DOI:** 10.2196/10949

**Published:** 2019-04-04

**Authors:** Daniel Clay Williams, Robert W Warren, Myla Ebeling, Annie L Andrews, Ronald J Teufel II

**Affiliations:** 1 Department of Pediatrics Medical University of South Carolina Charleston, SC United States

**Keywords:** electronic health records, user satisfaction, efficiency, physician survey

## Abstract

**Background:**

The effect electronic health record (EHR) implementation has on physician satisfaction and patient care remains unclear. A better understanding of physician perceptions of EHRs and factors that influence those perceptions is needed to improve the physician and patient experience when using EHRs.

**Objective:**

The objective of this study was to determine provider and clinical practice factors associated with physician EHR satisfaction and perception of patient impact.

**Methods:**

We surveyed a random sample of physicians, including residents and fellows, at a US quaternary care academic hospital from February to March 2016. The survey assessed provider demographics, clinical practice factors (ie, attending, fellow, or resident), and overall EHR experience. The primary outcomes assessed were provider satisfaction and provider perceptions of impact to patient care. Responses on the satisfaction and patient impact questions were recorded on a continuous scale initially anchored at neutral (scale range 0 to 100: 0 defined as “extremely negatively” and 100 as “extremely positively”). Independent variables assessed included demographic and clinical practice factors, including perceived efficiency in using the EHR. One-way analysis of variance or the Kruskal-Wallis test was used for bivariate comparisons, and linear regression was used for multivariable modeling.

**Results:**

Of 157 physicians, 111 (70.7%) completed the survey; 51.4% (57/111) of the respondents were attending physicians, and of those, 71.9% (41/57) reported a >50% clinical full-time-equivalency and half reported supervising residents >50% of the time. A total of 50.5% (56/111) of the respondents were primary care practitioners, previous EHR experience was evenly distributed, and 12.6% (14/111) of the total sample were EHR super-users. Responses to how our current EHR affects satisfaction were rated above the neutral survey anchor point (mean 58 [SD 22]), as were their perceptions as to how the EHR impacts the patient (mean 61 [SD 18]). In bivariate comparisons, only physician age, clinical role (resident, fellow, or attending), and perceived efficiency were associated with EHR satisfaction. In the linear regression models, physicians with higher reported perceived efficiency reported higher overall satisfaction and patient impact after controlling for other variables in the model.

**Conclusions:**

Physician satisfaction with EHRs and their perception of its impact on clinical care were generally positive, but physician characteristics, greater age, and attending level were associated with worse EHR satisfaction. Perceived efficiency is the factor most associated with physician satisfaction with EHRs when controlling for other factors. Understanding physician perceptions of EHRs may allow targeting of technology resources to ensure efficiency and satisfaction with EHR system use during clinical care.

## Introduction

Electronic health record (EHR) systems have been widely adopted in US hospitals in part due to financial incentive programs as well as the anticipated benefits of cost savings and improvements in safety and quality through a comprehensive approach to patient care [1-5]. Studies evaluating cost savings among hospitals after EHR adoption have had inconsistent findings [2,6]. In addition to potential cost savings, EHRs feature designs to improve patient safety through a variety of mechanisms such as real-time prompts during patient care encounters on drug dosing and potential medication error alerts [7-11]. While studies have shown that these prompts can prevent errors, they have also resulted in unintended consequences among providers such as alert fatigue [9-14].

Public perception of EHRs is generally positive but work by Emani et al [15,16] suggests physician skepticism might exist regarding the effect of meaningful use of EHRs on quality of care, patient-centeredness of care, and the patient care they personally provided. Although other studies regarding physician perceptions suggest EHRs may improve billing and quality, they also demonstrated skepticism regarding the impact on physician job satisfaction [17,18]. Decreased physician job satisfaction can lead to burnout, physician turnover, increased cost of physician recruitment, and potentially declines in quality of care [19-22]. While these studies have added to what is known, the lower response rates and focus on anticipated experience with meaningful use of EHR limits their generalizability and applicability to current provider practice.

The objective of this study was to determine provider and clinical practice factors, including perceived efficiency using the EHR, associated with physician EHR satisfaction and the perception of the EHR’s impact on patient care. Ultimately, gaining insight into this complex issue may inform future efforts to improve physician efficiency and satisfaction with EHRs and optimize the positive impacts on patient safety and quality of care.

## Methods

### Setting

This descriptive survey study took place at the Medical University of South Carolina (MUSC) from February to March 2016. MUSC is a 700-bed quaternary care academic hospital that includes all adult and pediatric primary care and subspecialty services. MUSC Health manages over 1,000,000 outpatient encounters and 40,000 admissions annually through the employment of approximately 1200 physicians and 700 residents and fellows in 25 clinical departments.

MUSC currently uses Epic EHR software (Epic Systems Corporation), having adopted Epic outpatient systems in May 2012 and full Epic Enterprise, including all components of the fully integrated Epic health system, in June 2014. Initially developed in 1979, Epic is currently one of the most widely used EHR software systems worldwide. Epic is a fully integrated and encompassing EHR through which all health-related information is shared at MUSC. At MUSC, all physicians (attendings, residents, and fellows) are required to complete 8 hours of Epic training in a simulated practice environment prior to initial credentialing. During the implementation phase of the EHR, each clinical area had defined physician super-users, who were engaged in ongoing monthly interactive meetings with the Epic build team to stay up to date on relevant changes and new training updates, to help with immediate clinical and EHR needs of their respective areas.

### Survey Assessment Tool

A team of EHR, clinical, and research experts (DW, RW, RJT) developed the survey content after a review of pertinent literature and key informant interviews with local stakeholders. The team piloted the survey for question clarity among a group of hospitalist physicians (n=8) and information technology medical directors that included physicians from a variety of pediatric and adult subspecialties (n=10). There were no content changes resulting from piloting, but several questions were clarified based on feedback (see Multimedia Appendix 1).

### Respondent Sampling

We used a random number generator to identify a random sample of 157 physicians from a master list of all MUSC providers. The quantitative data analyzed for this project was part of a larger EHR satisfaction assessment project at the university that also included qualitative analysis of physician interviews. The qualitative interview data is not included in this analysis. Ten information technology medical directors were tasked with the entire project; thus, the final sample size was selected based on the ability of these 10 physicians to collect the data including completion of a face-to-face interview about the current EHR product. We used the qualitative data from the face-to-face interviews to drive improvement processes at the institution; we did not use the qualitative data for this analysis. We used Research Electronic Data Capture (REDCap) (REDCap Consortium) software for survey administration and data collection. We distributed surveys by email through REDCap, and the responses remained anonymous. Nonrespondents received email reminders from area specific medical directors. We did not incentivize or distribute reimbursements for survey completion. Our institution’s institutional review board considered this project quality improvement.

### Primary Outcomes

The primary outcomes assessed were provider satisfaction and provider perceived impact to patient care. We assessed provider satisfaction through the question, “How does Epic affect you overall?” We assessed impact to patient care through the question, “How does Epic affect your patients overall?” We recorded both question responses on a continuous scale ranging from 0 to 100 with 0 labeled as “extremely negatively” and 100 labeled as “extremely positively.” We anchored the slide for the response initially at a neutral value (50), and the survey respondents modified the answer from there.

### Independent Variables

We assessed independent variables including physician demographics (age) and clinical practice factors. Clinical practice factors included clinical role (attending, resident, fellow), specialty department, percentage of clinical effort (reported clinical full-time-equivalent or cFTE), and percentage of attending providers whose encounters involved working with a trainee.

We also assessed perceived efficiency in using the EHR. We evaluated perceived provider efficiency through the statement, “Please rate your efficiency using Epic.” Responses were recorded on a continuous scale from 0 to 100 with 0 labeled as “extremely inefficient” and 100 labeled as “extremely efficient.” We anchored the slide for the response initially at a neutral value (50), and the survey respondents modified the answer from there.

The survey also evaluated physician EHR use experience (any EHR experience, any Epic experience, and Epic experience at MUSC in years of use), number of applications used in Epic, and Epic training above the standard eight hours (training as an Epic super-user).

### Analysis Plan

We completed bivariate comparisons using one-way analysis of variance, and we calculated Pearson correlation coefficients for the continuous independent variables (perceived efficiency) for both outcome variables (satisfaction and perceived patient impact). The team also created linear regression models to predict reported provider satisfaction and perceived patient impact. A secondary analysis of factors associated with perceived efficiency was completed using one-way analysis of variance. All analyses were completed using SAS statistical software (SAS Institute).

## Results

Of 157 randomly selected physicians, 111 (70.7%) completed the survey. An initial sample size of 160 was selected as described; however, 3 of the physicians randomly selected from the database were unable to respond due to temporary leave of absence (n=1) and recent retirement (n=2). A total of 51.3% (57/111) of the respondents were attending physicians, 32.4% (36/111) were residents, and 16.2% (18/111) were fellows. Mean age was 40.9 years (range 26 to 75 years) (Table 1). The mean age of the sample was similar to the mean age of all physicians at MUSC (39.8 years). A total of 50.5% (56/111) of the respondents were primary care practitioners.

**Table 1:** Survey respondent demographics (n=111).

Categorical variables				Value, n (%)
**Age in years**				
	20-29			19 (17.1)
	30-39			47 (42.3)
	40-49			18 (16.2)
	>50			27 (24.3)
**Clinical role**				
	**Attending**			57 (51.4)
		**Clinical full-time-equivalent**		
			<0.5	16 (28.1)
			0.5-0.99	13 (22.8)
			1	28 (49.1)
		**Percentage of time supervising residents**		
			<50	29 (50.9)
			51-99	18 (31.6)
			100	10 (17.5)
		**Super-user**		
			No	45 (78.9)
			Yes	12 (21.1)
	Fellow			18 (16.2)
	Resident			36 (32.4)
	**Postgraduate year (fellows and residents only; n=54)**			
		1		8 (14.8)
		2		11 (20.3)
		3		6 (11.1)
		4		12 (22.2)
		5		9 (16.7)
		6		5 (9.3)
		7		3 (5.6)
**Clinical department**				
	Anesthesia			5 (4.5)
	General medicine			34 (30.6)
	Pediatrics			22 (19.8)
	Psychiatry			11 (9.9)
	Radiology			7 (6.3)
	Medical subspecialty			21 (18.9)
	Surgical subspecialty			11 (9.9)
**Electronic health record experience in years**				
	<1			2 (1.8)
	1-5			38 (34.2)
	5-10			42 (37.8)
	>10			29 (26.1)


**Epic experience in years**				
	<1			21 (18.9)
	1-5			74 (66.7)
	5-10			16 (14.4)
**Epic experience at MUSC^a^ in years**				
	<1			19 (17.1)
	1-5			86 (77.5)
	5-10			6 (5.4)
**Number of systems used**				
	1			22 (19.8)
	2			52 (46.8)
	3			29 (26.1)
	4			8 (7.2)

^a^MUSC: Medical University of South Carolina.

The overall mean response to the question assessing physician satisfaction demonstrated that physicians were generally satisfied (mean 58 [SD 22]), especially in light of the question being anchored at a neutral response of 50. Overall, physicians also felt that the EHR has a positive impact on the patient experience (mean 61 [SD 18]).

In the bivariate comparisons assessing categorical independent variables, only physician age and clinical role (resident, fellow, attending) were associated with satisfaction, with older age and attending role reporting lower satisfaction scores (both *P*<.05; Table 2).

**Table 2:** Bivariate analysis for the primary outcome variables provider satisfaction (EHR affects you) and patient impact (EHR affects patients). Numerical value represents the mean score for each group.

Categorical variables				Satisfaction mean	*P* value	Patient impact mean	*P* value
Total population score				57.8		60.6	
**Age in years**					**.05**		**.27**
	20-29			67.5		65.8	
	30-39			59.3		60.7	
	40-49			49.1		54.4	
	>50			54.2		60.8	
**Clinical role**					**.01**		**.21**
	**Attending (n=57)**			52.1		57.9	
		**Clinical full-time-equivalent**			**.88**		**.57**
			<0.5	54.5		61.7	
			0.5-0.99	50.9		58.3	
			1.0	51.2		55.6	
		**Percentage of time supervising residents**			**.07**		**.24**
			<50	**52.9**		**61.4**	
			51-99	44.0		52.2	
			100	64.3		57.9	
		**Super-user**			**.66**		**.39**
			No	52.4		55.9	
			Yes	50.8		65.3	
	Fellow			66.7		65.6	
	Resident			62.5		62.3	
	**Postgraduate year (fellows and residents only)**				**.18**		**.37**
		1		63.0		57.5	
		2		76.1		70.7	
		3		59.2		62.7	
		4		57.4		61.0	
		5		57.1		57.9	
		6		63.8		64.4	
		7		77.0		78.0	
**Clinical department**					**.22**		**.26**
	Anesthesia			70.2		63.8	
	General medicine			58.1		63.5	
	Pediatrics			58.7		60.0	
	Psychiatry			62.0		59.2	
	Radiology			62.4		59.6	
	Medical subspecialty			47.1		52.7	
	Surgical subspecialty			62.8		68.5	
**Electronic health record experience in years**					**.11**		**.36**
	<1			84.0		82.0	
	1-5			61.7		59.3	
	5-10			56.5		61.2	
	>10			52.8		59.9	
**Epic experience in years**					**.12**		**.42**
	<1			66.5		64.6	
	1-5			55.8		59.1	
	5-10			55.7		62.2	
**Epic experience at MUSC^a^** **in years**					**.27**		**.47**
	<1			64.1		60.4	
	1-5			57.0		61.2	
	5-10			49.2		52.0	
**Number of systems used**					**.51**		**.15**
	1			60.4		59.3	
	2			57.6		62.6	
	3			54.0		55.4	
	4			65.8		69.4	

^a^MUSC: Medical University of South Carolina.

**Figure 1:**
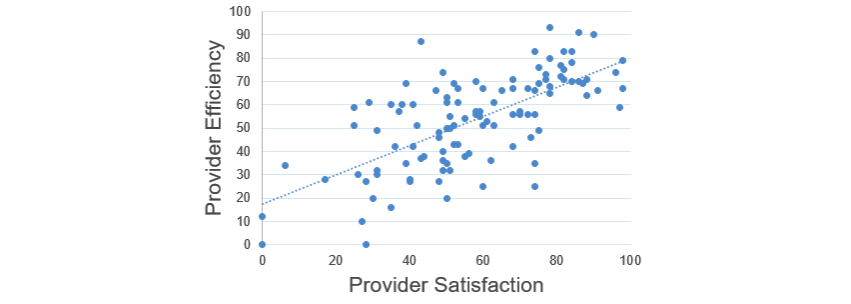
Scatter plots demonstrating the positive association between provider efficiency and satisfaction (*r*=0.68).

**Figure 2:**
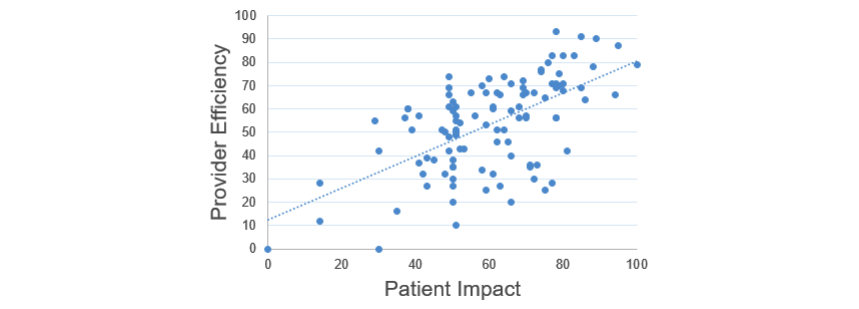
Scatter plot demonstrating the positive association between provider efficiency and provider reported patient impact (*r*=0.6).

None of the assessed categorical variables were associated with perceived patient impact; however, physician reported perceived efficiency was correlated with both provider satisfaction (*r*=0.68) and perceived patient impact (*r*=0.6; Figures 1 and 2), indicating that physicians reporting higher perceived efficiency also reported higher overall satisfaction and EHRs having an overall more positive impact on the patient.

The regression model for physician satisfaction with predictors including clinical role, experience, and efficiency produced an *R*^2^ of 0.5. As seen in Table 3, only perceived efficiency had a significant positive regression weight indicating that physicians with higher reported efficiency also reported higher overall satisfaction after controlling for other variables in the model. For every 1-point increase in efficiency, satisfaction scores increase by 0.74.

**Table 3:** Linear regression table predicting physician satisfaction: *R*^2^=0.5; *P*<.001.

Variable	Parameter estimate	Standard error	*t* value	*P* value
Intercept	19.9	6.6	3.0	.003
Clinical role	1.7	1.8	1.0	.34
Epic experience at MUSC^a^	–0.7	0.8	0.8	.39
Efficiency	0.7	0.1	9.1	<.001

^a^MUSC: Medical University of South Carolina.

The regression model for physician-reported perceived patient impact, which also included clinical role, experience, and perceived efficiency as predictors, produced an *R*^2^ of 0.4. As seen in Table 4, only perceived efficiency had a significant positive regression weight indicating that physicians with higher reported efficiency also reported higher positive perceived patient impact after controlling for other variables in the model. For every 1-point increase in efficiency, perception of patient impact increases by 0.53.

Because reported efficiency was the factor most predictive of both physician satisfaction and perceived patient impact, we conducted a secondary analysis of physician reported perceived efficiency. Overall, physician responses in rating their personal efficiency using EHR were positive (mean 54 [SD 20]). In bivariate comparisons, only clinical role was associated with perceived efficiency, with attending physicians reporting lower efficiency (Table 5).

**Table 4:** Linear regression table predicting perceived patient impact: *R*^2^=0.4; *P*<.001.

Variable	Parameter estimate	Standard error	*t value*	*P value*
Intercept	32.00	5.90	5.40	<.001
Clinical role	–0.30	1.60	–0.20	.87
Epic experience at MUSC^a^	0.05	0.70	0.07	.94
Efficiency	0.53	0.07	7.70	<.001

^a^MUSC: Medical University of South Carolina.

**Table 5:** Bivariate analysis for factors associated with perceived efficiency.

Categorical variables				Efficiency, mean	*P* value
Total population perceived efficiency				53.9	
**Age in years**					.11
	20-29			60.7	
	30-39			56.2	
	40-49			50.1	
	>50			47.8	
**Clinical role**					.01
	**Attending**			48.5	
		**Clinical full-time-equivalent**			.28
			<0.5	55.8	
			0.5-0.99	46.6	
			1.0	45.1	
		**Percentage of time supervising residents**			.08
			<50	48.6	
			51-99	41.2	
			100	60.7	
		**Super-user**			.37
			No	46.9	
			Yes	54.3	
	Fellow			63.8	
	Resident			57.6	
	**Postgraduate year (fellows and residents only)**				.05
		1		53.1	
		2		66.6	
		3		56.5	
		4		57.8	
		5		52.7	
		6		61.0	
		7		85.0	
**Clinical department**					.86
	Anesthesia			54.2	
	General medicine			53.0	
	Pediatrics			58.0	
	Psychiatry			56.8	
	Radiology			58.0	
	Medical subspecialty			49.7	
	Surgical subspecialty			50.9	
**Electronic health record experience in years**					.12
	<1			83.0	
	1-5			56.0	
	5-10			53.6	
	>10			49.8	
**Epic experience in years**					.17
	<1			60.1	
	1-5			51.4	
	5-10			57.3	
**Epic experience at MUSC^a^** **in years**					.47
	<1			58.7	
	1-5			53.2	
	5-10			48.8	
**Number of systems used**					.67
	1			49.7	
	2			55.2	
	3			55.7	
	4			50.9	

^a^MUSC: Medical University of South Carolina.

## Discussion

### Principal Findings

This survey of physicians practicing at all levels of training and experience at a large academic medical center with a fully integrated and established EHR reveals that the EHR has had an overall positive influence on physician satisfaction with the EHR and perceived positive influence on patient care. Previous studies have reported physician concerns and challenges with provider use of an EHR system, such as those found in reports from Emani et al [15,16] and Shanafelt et al [20]. A 2012 study by Heyward et al [23] surveyed community-based clinicians before and after EHR implementation and found decreasing rates of overall job satisfaction among its providers. Our findings, in contrast to these reports, showed clinicians in a variety of clinical settings and practice types rated satisfaction with our EHR system positively and felt it has a positive impact on the care they provide.

Although older and attending-level physicians appeared more likely to report decreased satisfaction with EHR in our bivariate comparisons, it was their own perceived efficiency in using the EHR that was predictive of both satisfaction and positive impact for patients in adjusted analysis. We assessed perceived efficiency in the survey with the question, “Please rate your efficiency using Epic.” With perceived efficiency demonstrating the strongest association with physician satisfaction, we felt a more in-depth assessment of factors that influence perceived efficiency was needed. In a second bivariate analysis using efficiency as an outcome, only clinical role was associated with perceived efficiency. Attending physicians, when compared to residents and fellows, had the lowest overall perceived efficiency. This difference in perception of efficiency is likely multifactorial but could represent a true difference in how efficient attending-level providers are in using the EHR compared to their peers. To our knowledge, no validated measure exists to compare actual use efficiency.

Our diverse, randomly selected sample enables insight into perceptions of providers across disciplines and at various levels of training and EHR experience. For example, attending physicians and those practicing for a longer period of time may have had more experience with non-EHR systems and therefore may have a different perspective on EHR impact on patient care. Additionally, younger providers may have more general experience with technology. This experience may enable them to adapt easily to EHR adoptions, updates, or modifications.

We identified a trend *(P*=.06) in bivariate analysis toward increased time spent supervising residents with higher reported EHR satisfaction, but this was not significant. Attending physicians who supervise residents have less direct EHR use and responsibility. For example, physicians responsible for supervising residents more commonly cosign documentation as compared to writing notes and entering orders directly. Future studies may be needed to further evaluate the impact of resident supervision on attending use and satisfaction with EHRs.

### Limitations

Our study has limitations. Although our response rate to the survey was above a commonly accepted benchmark (60%), it was only distributed at a single center. Although the EHR system used at this center is one of the most commonly adopted EHRs in the United States, it does have some degree of customizability and therefore our results may not be generalizable. Additionally, we chose our sample based on the number of surveys felt to be feasible to perform (N=160), and some of our associations that were close to significant may have become significant with a larger sample. This impact may have been greater when investigating subgroups such as attending only. Furthermore, our study assesses association and not causations. Due to the desire to keep the survey brief, other EHR-specific factors influencing satisfaction and clinician perception of the EHR’s impact on patient care were not assessed. This represents a potential area for further research. It is helpful that EHR implementation occurred between May 2012 and June 2014 as many of the surveyed providers did have experience caring for patients without an EHR, but we did not measure important variables like efficiency before and after implementation as would be required to test causation. Last, further work is also needed to determine if perception of poor efficiency is correlated with actual efficiency. Unfortunately, no standard measure for efficiency exists with EHRs.

### Conclusions

In our diverse sample of providers, perceived efficiency in using the institution’s EHR was the factor most associated with both satisfaction and perceived impact to patient care. Targeting at-risk groups for training, efficiency improvement efforts, and continued monitoring especially during major upgrades may be needed to improve efficiency as a way to increase physician satisfaction and ensure high-quality patient care.

## Appendix

Multimedia Appendix 1 Clinician survey tool.

## Abbreviations

cFTE: clinical full-time-equivalent
EHR: electronic health record
MUSC: Medical University of South Carolina
REDCap: Research Electronic Data Capture
