# Supplementary material for: Physician Use of Electronic Health Records: Survey Study Assessing Factors Associated With Provider Reported Satisfaction and Perceived Patient Impact
Source: JMIR Med Inform. 2019 Apr 4;7(2):e10949. doi: 10.2196/10949 (PMC6470463; doi:10.2196/10949)
Supplement: Multimedia Appendix 1 [file medinform_v7i2e10949_app1.pdf]

Current Role: Resident/Fellow/Attending (circle)

Years in Current Role:
- PGY#:
- Years as attending:

How many years have you used the following (include years as a student, resident, fellow, attending)?
- Any EHR (Practice Partner, Epic, Oacis, Mckesson, etc):
- EPIC total (outside and at MUSC):
- EPIC at MUSC:

What “parts” of Epic do you use at MUSC (select all that apply)?
- ○ Inpatient
- ○ Outpatient
- ○ ASAP (Emergency Department)
- ○ Optime/Anesthesia (Operating room)
- ○ Radiant (radiologists only)
- ○ Stork (labor and delivery)
- ○ Kaleidoscope (ophthalmology)
- ○ Beacon (oncology)

How does Epic affect your patients overall?

Extremely Negatively — Extremely Positively

How does Epic affect you overall?

Extremely Negatively — Extremely Positively

Please rate your efficiency using Epic.

Extremely Inefficient — Extremely Efficient

Please rate your efficiency compared to your peers (those in the same specialty/subspecialty using Epic).

Extremely Less Efficient — Extremely More Efficient

Describe the most positive effect Epic has on your patients.	
Describe the most negative effect Epic has on your patients.	
Describe the most positive effect Epic has on you.	
Describe the most negative effect Epic has on you.	
